# Longitudinal study of changes in γδ T cells and CD4^+^ T cells upon asymptomatic malaria infection in Indonesian children

**DOI:** 10.1038/s41598-017-09099-z

**Published:** 2017-08-18

**Authors:** Sanne E. de Jong, Vera E. R. Asscher, Linda J. Wammes, Aprilianto E. Wiria, Firdaus Hamid, Erliyani Sartono, Taniawati Supali, Hermelijn H. Smits, Adrian J. F. Luty, Maria Yazdanbakhsh

**Affiliations:** 1 Leiden Immunoparasitology Group, Department of Parasitology, Leiden University Medical Center, Albinusdreef 2, 2333 ZA Leiden, The Netherlands; 2 Department of Parasitology, University of Indonesia, Jl. Salemba Raya No. 6, 10430 Jakarta Pusat, Indonesia; 3 Department of Microbiology, Hasanuddin University, Jl. Perintis Kemerdekaan, Km. 10, 90245 Makassar, Indonesia; 4 Mère et Enfant Face aux Infections Tropicales, UMR 216, French National Research Institute for Sustainable Development (IRD), Paris, France; 5 Faculty of Pharmaceutical and Biological Sciences of Paris, Paris Descartes University, Paris, France; 6 Department of Medical Microbiology & Infectious Diseases, Erasmus MC, Wytemaweg 80, 3015 CN Rotterdam, The Netherlands

## Abstract

Both γδ T cells and CD4^+^ T cells have been implicated in immunity to malaria, but their association with natural gain or loss of infection has not been studied before. Therefore, we followed up asymptomatic children living in an area endemic for malaria in Indonesia for 21 months. The percentage of γδ T cells was related to both current and previous infection, with higher percentages in infected than uninfected children and declining after infections resolve. Infected children also had higher levels of Th1 and Th17 cells, lower levels of CD25^Hi^ FOXP3^+^ regulatory T cells (Tregs), but similar levels of Th2 cells as compared to uninfected children. However, TNF, IFN-γ, and IL-17 cytokine responses to *Plasmodium falciparum*-infected red blood cells (PfRBCs) were similar, while IL-5 and IL-13 responses were lower in infected children. Furthermore, infected children had more phenotypically exhausted PD-1^+^ CD4^+^ T cells, more Tregs expressing TNF-RII, and higher IL-10 responses to PfRBCs, which persisted following resolution of infection. Altogether, this study demonstrates that asymptomatic malaria infection is associated with some long-lasting changes in the frequencies and immunoregulation of circulating innate and adaptive T cells, which might in part explain how pre-exposure to malaria affects responses to subsequent immunological challenges.

## Introduction

In areas where malaria is endemic, immune responses can develop upon repeated exposure that lead not only to control of parasites, but also symptoms^1^. Moreover, repeated malaria exposure is believed to result in compromised responses to vaccines^2^. However, neither protective immune responses nor regulatory responses are fully understood. Both γδ T cells and CD4^+^ T cells have been implicated in immunity and tolerance to malaria.

γδ T cells are thought to participate in the innate immune response to malaria infection, for example by producing pro-inflammatory cytokines such as IFN-γ and TNF, or by killing blood-stage merozoites directly with cytotoxic granulysin^3–6^. Next to limiting malaria parasites^4^, changes in γδ T cells might also be involved in the modulation of inflammation during malaria infection^5^. Most studies of the association between malaria and γδ T cells have been based on cross-sectional analysis of clinical cases versus controls, with little data on the behaviour of these cells longitudinally in asymptomatic subjects.

Another group of cells studied extensively in malaria, the CD4^+^ T cells, play a central role in immunity to malaria parasites. In particular Th1 cells have been described to play an important role in limiting infection^7^, while expression of regulatory T cells (Tregs) as well as regulatory molecules might play a pivotal role in ensuring a balanced response whereby inflammation is kept under control^8^. With respect to the latter, altered levels of PD-1^+^ (CD279) CD4^+^ T cells and Tregs have been found in people with acute malaria or in chronically-exposed subjects^9–11^. Moreover, increased expression of TNF-RII (CD120b) on Tregs, which is critical for their stability and function, is associated with malaria infection^12, 13^. However, as for γδ T cells, most studies examining CD4^+^ T cells and their phenotype has been cross-sectional in nature.

In this study, we investigate the role of γδ T cells and CD4^+^ T cells in immune responses during natural malaria infection. Asymptomatic infected and uninfected Indonesian schoolchildren were followed up over time and cell percentages, cytokine responses, and immunoregulation were monitored.

## Results

### Study population

123 Indonesian children were followed up for 21 months. Data was available from 77 of these children at 9 months and from 115 of these children at 21 months. At baseline, 22.0% of the children were asymptomatically infected with *Plasmodium*, of which 66.7% carried *P*. *falciparum*, 33.3% *P*. *vivax*, and *11*.*1% P*. *malariae* (including co-infections with two species) based on PCR diagnosis (Supplementary Table S1). The prevalence remained at 22.1% after 9 months and decreased to 8.7% after 21 months, which was ascribed to reinforcement of malaria control by the local health centre. Next to malaria infection, 93.3% of all children at baseline was infected with one or more helminth species (Supplementary Table S2). Helminth prevalence decreased to 69.6% after 9 months and to 74.3% after 21 months, and was comparable between the children with and without malaria infection. Sex ratios (49% male) and body mass index (mean 14.6 kg/m^2^, −1.1 zBMI) were comparable between asymptomatic *Plasmodium* infected and uninfected children, although the infected children were slightly older (mean 9.4 vs. 8.3 years old, P = 0.024).

### Asymptomatic infected children have relatively more γδ T cells, Th1 cells, and Th17 cells, but comparable or downregulated cytokine responses to PfRBC stimulation

**Figure 1:**
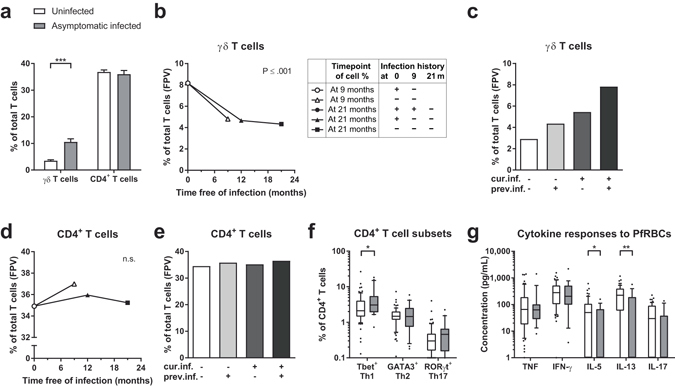
Profiles of γδ T cells and CD4^+^ T cells. (**a**) The percentage of γδ T cells and CD4^+^ T cells amongst total T cells compared between uninfected and asymptomatic infected children at baseline. Bar graphs show means and standard errors of the mean (SEM). (**b**,**d**) The percentage of γδ T cells and CD4^+^ T cells in children uninfected at either the 9 (open symbols) or 21 months’ (closed symbols) time point plotted against the time period these children were found to be free of infection. Data shown are fixed predicted values (FPVs) of linear mixed modelling. The legend shows the malaria infection status at baseline (0 months), and after 9 and 21 months of follow-up; + indicates infected and - indicates uninfected. The statistical significance of the change in cell percentage over time is indicated by the P value. (**c**,**e**) The percentage of γδ T cells and CD4^+^ T cells amongst total T cells compared between currently infected (cur.inf.) and/or previously infected (prev.inf.) children, *i*.*e*. recently and longer-time infected and uninfected children within the study period. (**f**) The percentage of Th1, Th2, and Th17 subsets amongst CD4^+^ T cells compared between uninfected and asymptomatic infected children at baseline. Box plots show medians and 10–90% whiskers. (**g**) TNF, IFN-γ, IL-5, IL-13, and IL-17 cytokine responses of PBMCs to PfRBC stimulation, after background subtraction of responses to uninfected RBC, compared between uninfected and asymptomatic infected children at baseline. * indicates analysis with independent-samples T test (bar graphs) or Mann-Whitney U test (box plots) of baseline data. Analysis with linear mixed models of all time points together is shown in Supplementary Figure S1. *P ≤ 0.05. **P ≤ 0.01. ***P ≤ 0.001.

To assess the dynamic changes in γδ T cells and CD4^+^ T cells in response to malaria infection, asymptomatic *Plasmodium* infected and uninfected children were compared during a 21-month follow-up. Infected children had higher percentages of γδ T cells at baseline and during follow-up as analysed by linear mixed modelling (Fig. 1a and Supplementary Figs S1a and S2a). These higher levels decreased after resolution of infection as shown in Fig. 1b (raw data in Supplementary Fig. S3a). Linear mixed modelling predicted that children who had been free of infection for 9 months (negative at baseline and at 9 months, indicated by ∆) had lower γδ T cell levels at the 9-month time point than children who had recently lost their infection (positive at baseline, negative at 9 months, indicated by ○) (8.2% vs. 4.8%). Regarding γδ T cell level predictions for the 21-month time point, children who had been free of infection for 12 months (positive at baseline, negative at 9 months and 21 months, indicated by ▲) or for 21 months (negative at baseline, 9 months, and 21 months, indicated by ■) had lower γδ T cell levels than children who had recently lost their infection (positive at baseline and 9 months, negative at 21 months, indicated by ●). Another linear mixed model revealed that children who had remained free of infection throughout the study period had the lowest levels as well, and showed in addition that those found to be infected at consecutive time points had the highest percentages of γδ T cells, *i*.*e*. higher than newly infected children (Fig. 1c). This indicates that the presence of malaria infection is associated with rising γδ T cells percentages, which wane when the infection is cleared, but leaves at least a temporary footprint of a higher γδ T cell percentage compared to those who have been free of infection.

**Figure 2:**
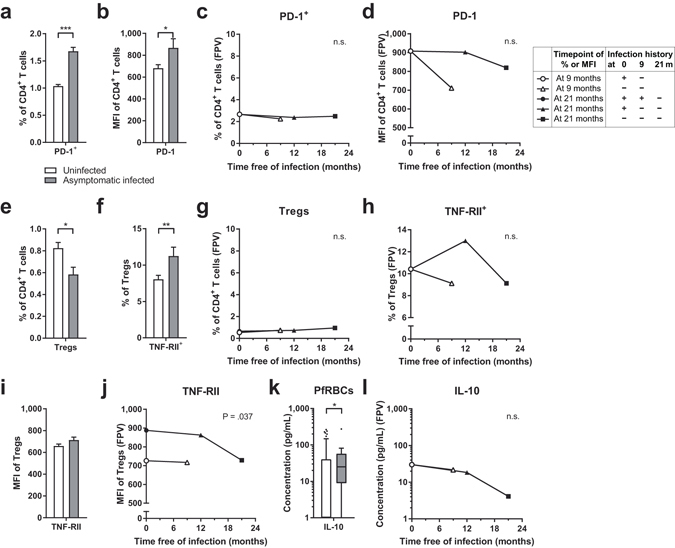
Profiles of PD-1^+^ CD4^+^ T cells and Tregs and IL-10 production. (**a**,**b**,**e**,**f**,**i**) The percentage of cells or the expression level of markers on cells compared between uninfected and asymptomatic infected children at baseline. The expression levels are based on geomean fluorescent intensities (MFI). Bar graphs show means and standard errors of the mean. (**c**,**d**,**g**,**h**,**j**) The percentage of cells or the expression level of markers on cells in children uninfected at either the 9 or 21 months’ time point, plotted against the time period these children had been free of infection (see description Fig. 1). (**k**) IL-10 cytokine production by PBMCs in supernatant in response to *in vitro* PfRBC stimulation, after background subtraction of responses to uninfected RBC. l) IL-10 cytokine production in children uninfected at either the 9 or 21 months’ time point, plotted against the time period these children had been uninfected.

When comparing the percentage of total CD4^+^ T cells, there was no significant difference between infected and uninfected children at baseline or during follow-up, neither a change between time points after resolution of infection (Fig. 1a, d, and e and Supplementary Figs S1b, S2b, and S3b). However, the CD4^+^ T cell subset composition was altered by infection. The percentage of T-bet^+^ (Th1) and RORγt^+^ (Th17) CD4^+^ T cells was higher in infected children than in uninfected children, either at baseline or during follow-up (Fig. 2f and Supplementary Figs S1c and S2c). However, unlike γδ T cells, these levels did not decrease after resolution of infection within the study period (Supplementary Figs S3g, S3h, and S4a). Therefore, malaria infection seems to result in a long-lasting increase of Th1 and Th17 cells. The percentage of GATA3^+^ (Th2) CD4^+^ T cells was not associated with a change in infection status.

We subsequently compared peripheral blood mononuclear cell (PBMC) cytokine responses to *in vitro* stimulation with *P*. *falciparum*-infected red blood cells (PfRBC) between infected and uninfected children, but found no significant differences for TNF, IFN-γ, and IL-17 levels in supernatant at any study time point (Fig. 1g and Supplementary Figs S1d, S2d, and S4b). IFN-γ is the hallmark cytokine for Th1 cells and IL-17 for Th17 cells, while TNF can be produced by both CD4^+^ T cell subsets and all three cytokines can be produced by γδ T cells. Nevertheless, the relatively high levels of γδ T cells, Th1 cells, and Th17 cells in infected children were not reflected by higher cytokine responses to PfRBCs. Furthermore, despite similar levels of Th2 cells in infected and uninfected children, the Th2 cytokines IL-5 and IL-13 were lower in infected children than in uninfected children at baseline and during follow-up, suggesting downregulation of Th2 responses in infected children (Fig. 1g and Supplementary Figs S1d, S2d, S3i, S3j, and S4b).

### Higher CD4^+^ T cell exhaustion, Treg activation, and IL-10 responses are associated with malaria

To investigate the effects of malaria infection on immunoregulation amongst CD4^+^ T cells, we assessed expression of the exhaustion marker PD-1 on CD4^+^ T cells, Treg levels, and IL-10 cytokine responses to PfRBCs in asymptomatic infected and uninfected children. If altered by malaria infection, this might explain why the higher percentages of Th1, Th17, and γδ T cells in infected children were not accompanied by enhanced cytokine responses and how the Th2 responses were downregulated.

To begin with, infected children had more phenotypically exhausted CD4^+^ T cells at baseline and during follow-up than uninfected children, based both on the percentage of PD-1^+^ cells and on the expression level of PD-1 on CD4^+^ T cells (Fig. 2a and b and Supplementary Figs S1e and S2e). The exhausted phenotype was long-lasting, as the expression did not decrease after resolution of malaria infection (Fig. 2c and d and Supplementary Fig. S3c and d).

Furthermore, in infected children, the percentage of CD25^Hi^ FOXP3^+^ CD4^+^ Tregs was lower at baseline and during follow-up than in uninfected children (Fig. 2e and Supplementary Figs S1f and S2f). Despite lower Treg levels, a higher percentage of these cells expressed TNF-RII in infected children (Fig. 2f and Supplementary Figs S1g and S2g), suggesting relatively more Tregs with higher suppressive capacity as compared to Tregs in uninfected children. These differences were long-lasting, as the percentage of Tregs or TNF-RII^+^ Tregs did not change significantly after resolution of infection (Fig. 2h and Supplementary Figs S3e and S3f). In addition, although not statistically significant, the expression level of TNF-RII on individual Tregs appeared to be higher in infected than uninfected children (Fig. 2i and Supplementary Figs S1h and S2h). However, after resolution of infection, the expression level of TNF-RII decreased significantly (Fig. 2j), suggesting that although the effects on Treg numbers were long-lasting, the expression level of TNF-RII on individual Tregs was only elevated during parasitemia. Thus, the expression of TNF-RII seems to be more dynamically associated with presence of malaria infection than Treg numbers.

At last, PBMCs of infected children produced more of the regulatory cytokine IL-10 in response to *in vitro* PfRBC stimulation than those of uninfected children, at baseline and during follow-up (Fig. 2k and Supplementary Figs S1i and S2i). As the IL-10 levels did not decrease significantly after resolution of infection, the increased IL-10 response to PfRBCs appeared long-lasting (Fig. 2l and Supplement S3k).

## Discussion

Our results demonstrate that the percentages of γδ T cells were higher in children presenting with asymptomatic infections than in uninfected children, and that these levels decreased after clearance of parasites. This is in line with other studies in Ethiopia^14^ and Thailand^15^ in infected adults who, although presenting with acute malaria, had higher levels of γδ T cells than healthy controls. In Ghanaian children and adults, γδ T cell numbers increased after admission for malaria and these levels appeared higher than those found in healthy controls^16, 17^. Also, controlled infection of malaria-naïve adults results in an increase in γδ T cells^18, 19^. With our longitudinal approach, we could also detect a tendency for increased γδ T cells in subjects with a history of malaria infection during the study period. Along with relatively more γδ T cells, a cell type capable of strong responses to PfRBCs^20^, higher cytokine levels after PfRBC stimulation might have been expected in infected children, but TNF, IFN-γ, and IL-17 responses were not significantly different from uninfected children.

Shifts among the Vδ1^+^ and Vδ2^+^ subsets of γδ T cells have been found to occur with malaria infection. In Ethiopian adults^14^ and Ghanaian children^17^, the increase in total γδ T cells as compared to healthy controls or upon malaria infection was mainly due to Vδ1^+^ cells. Furthermore, in Ugandan children, Vδ2^+^ cells decreased in numbers and showed decreased proliferation, cytokine production, and degranulation with repeated malaria exposure^5, 6^. Although we did not analyse these γδ T cell subsets separately, the increase in total γδ T cells in infected children in our study might also be due to Vδ1^+^ cells. As Vδ2^+^ but not Vδ1^+^ cells predominantly respond to *in vitro* PfRBC stimulation^5, 6^, an increase in Vδ1^+^ cells would not result in increased cytokine responses, which would be in line with our observations.

The percentages of Th1 and Th17 cells, but not of Th2 cells, were higher in infected than in uninfected children. This is in line with our previous study, where Th1 levels were higher and Th2 levels unaltered in asymptomatic infected Indonesian children as compared to uninfected children^12^. Despite these higher percentages of Th1 and Th17 cells in infected children, TNF, IFN-γ, and IL-17 cytokine responses to PfRBC stimulation were not significantly different between infected and uninfected children. Furthermore, despite the comparable Th2 cell percentages, IL-5 and IL-13 cytokine responses were lower in infected children. In our previous study, TNF, IFN-γ, and IL-13 were also analysed and gave similar results^12^. In Papua New Guinea, TNF, IFN-γ, and IL-4 cytokine responses to PfRBCs did not differ between infected and uninfected children^21^. Thus, IL-4 responses might differ from the other Th2 cytokines IL-5 and IL-13, or Th2 responses in general differed between our studies. In any case, the lack of enhanced TNF, IFN-γ, and IL-17 responses and the downregulated Th2 response in our current study might be related to the changes observed that are associated with immunoregulation amongst CD4^+^ T cells.

Indeed, asymptomatic infected children had relatively more phenotypically exhausted PD-1^+^ CD4^+^ T cells and produced more IL-10 in response to PfRBCs than uninfected children. In studies of children in Mali^22^ and Kenya^9^, malaria infection was also associated with PD-1^+^ CD4^+^ T cells. Furthermore, a recent study showed that PD-1^+^ CTLA4^+^ CD4^+^ T cells can coproduce IFN-γ and anti-inflammatory IL-10 in response to *Plasmodium* antigens, and can suppress proliferation of other CD4^+^ T cells after *P*. *falciparum*-specific and polyclonal stimulation in a contact-dependent manner^11^. Besides PD-1^+^ T cells and IL-10 responses, infected children in our study had a higher percentage of Tregs expressing TNF-RII, which indicates higher suppressive capacity^13^. This is in accordance with our previous study amongst Indonesian children, where TNF-RII expression was higher during infection and decreased upon treatment^12^. Thus, the higher percentages of PD-1^+^ T cells and TNF-RII on Tregs and the higher IL-10 responses might have affected cytokine production upon PfRBC stimulation, resulting in similar PfRBC-induced TNF, IFN-γ, and IL-17 production and reduced IL-5 and IL-13 production by cells from infected children as compared to uninfected children. Additional markers and functional studies are required to determine the separate contributions of these cells and cytokines to immunoregulation and to determine whether our PD-1^+^ CD4^+^ T cells were truly exhausted.

Although displaying higher TNF-RII levels, Tregs nevertheless represented a lower proportion of CD4^+^ T cells in asymptomatic infected children than in uninfected children. This is in line with two previous studies amongst Indonesian children^12, 23^, but in contrast to studies amongst Indonesian^23^ and Peruvian^24^ adults where Treg percentages were comparable between asymptomatic infected and uninfected subjects. The differences between these studies might relate to differences in age or degree of exposure, as a study amongst Ugandan children showed that repeated malaria exposure in regions with high levels of malaria transmission results in a decline of Treg percentages after a transient increase upon infection^25^.

Some of the differences between asymptomatic infected children and uninfected children were long-lasting, persisting after resolution of infection. For example, the higher levels of Th1 and Th17 cells and PD-1 on CD4^+^ T cells were long-lasting, as were the lower Treg levels, the higher number of Tregs expressing TNF-RII, and the higher IL-10 response. One interpretation could be that these profiles are enhanced by repeated malaria exposure and might thus be related to naturally acquired tolerance against symptomatic disease^26^.

Our findings shed new light on the dynamic changes of γδ T cells and CD4^+^ T cells during follow-up of asymptomatic children living in an area where malaria is endemic. Changes during asymptomatic malaria infection and following parasite clearance suggest short- and long-term effects of infection on γδ T cells and CD4^+^ T cells, as well as involvement of immunoregulation. The combination of these altered cellular immunological footprints could be intimately linked to asymptomatic carriage, immunity to malaria infection and disease, as well as responses to inflammatory insults. Studies involving larger sample sizes would be necessary to prove this.

## Methods

### Study population

The study described here is part of the ImmunoSPIN trial, which was conducted in the Ende district of Flores island in Indonesia^27–29^. This double-blind placebo-controlled trial was initiated in 2008 by randomising households in the coastal village Nangapanda to receive either a single dose of 400 mg of the anthelminthic drug albendazole or a matching placebo, every three months over a two-year period. At baseline, 9 months, and 21 months, venous blood was drawn from the participants for cellular assays and stored for determination of infection with *Plasmodium*. Included in the present analysis were a total of 123 school children, between 4 and 15 years old, with complete baseline data, and either asymptomatic *Plasmodium*-infected (including multiple *Plasmodium* species) or uninfected. Individuals with symptomatic malaria were treated according to the current guidelines at the local health centre, and not included in this study. Clearance of asymptomatic infection during the trial could have occurred naturally or due to self-sought medical treatment. The ImmunoSPIN trail was performed in accordance with guidelines approved by the Committee of Medical Research Ethics of the University of Indonesia, and written informed consent was obtained from all participants’ parents or guardians.

### Parasitology

*Plasmodium* infection was determined retrospectively by microscopy of blood slides and by quantitative real-time polymerase chain reaction (qPCR) of whole blood as described before^30^. *P*. *falciparum*, *P*. *vivax*, and *P*. *malariae* species were endemic; no children were infected with *P*. *ovale*. Helminth infection was determined by microscopy after formol-ether concentration for *Trichuris trichiura* and by multiplex qPCR for hookworm (*Ancylostoma duodenale* and *Necator americanus*), *Ascaris lumbricoides*, and *Strongyloides stercoralis* as described before^29^.

### Cell isolation and stimulation

PBMCs were obtained by gradient centrifugation of heparinised venous blood over Ficoll (Apotheek AZL, Leiden, the Netherlands) in the field laboratory in Nangapanda, Indonesia. A part of the PBMCs was fixed for flow cytometric analysis and a part was stimulated for analysis of cytokine responses. 0.4 × 10^6^ cells were stimulated in Nunc 96-well round bottom MicroWell plates with Nunclon delta surface (ThermoFisher Scientific, Waltham, MA, USA) in 200 µL 10% foetal bovine serum (FBS; Greiner Bio-One Frickenhausen, Germany)/RPMI 1640 medium (Invitrogen, Carlsbad, CA, USA) for 96 hours with 1 × 10^6^ PfRBCs, uninfected RBCs (uRBCs), or a medium control, after which supernatants were obtained.

### Cytokine measurements

Supernatants were kept at −20 °C and transported to Leiden, the Netherlands, where cytokine responses were quantified using Luminex cytokine kits (Biosource, Camarillo, CA, USA) and a Liquichip 200 Workstation (Qiagen, Venlo, the Netherlands) equipped with Liquichip analyser software (Qiagen, Venlo, the Netherlands). Cytokine levels below the assay’s detectable range were replaced by half the detection limit. The detection limits were 2.26 pg/mL for TNF, 0.68 pg/mL for IFN-γ, 0.16 pg/mL for IL-5, 0.44 pg/mL for IL-13, 0.60 pg/mL for IL-17, and 0.78 pg/mL for IL-10.

### Flow cytometry

PBMC were fixed and permeabilised with FOXP3 Fixation/permeabilisation buffer (eBioscience Inc., San Diego, CA, USA) for 1 hour at room temperature and cryopreserved in 10% FBS/10% dimethyl sulfoxide (DMSO; Merck Millipore)/RPMI at −20 °C. The samples were transported to Leiden, the Netherlands, where they were stored at −80 °C. The cells were thawed and stained for 30 minutes at 4 °C with the panel shown in Supplementary Table S3 in FOXP3 permeabilisation buffer (eBioscience) with FcγR-binding inhibitor (eBioscience; 100x diluted). The samples were measured with a BD FACSCanto II flow cytometer (BD Biosciences, San Jose, CA, USA), equipped with a blue (488 nm Coherent Sapphire solid state laser, 20 mW laser output), red (633 nm JDSU 1144P-3581 HeNe laser, 17 mW laser output) and violet (405 nm Qioptiq iFLEX2000 solid state diode, 30 mW fibre power output) laser and detectors arranged as in Supplementary Table S4. The data was compensated with BD CompBeads in BD FACSDiva 6 (BD Biosciences) and analysed with FlowJo 9 (FlowJo LLC, Ashland, OR, USA). The gating strategy is shown in Supplementary Figure S5.

### Statistical analysis

Statistical analysis was performed in IBM SPSS Statistics version 21 for Windows (IBM Corp., Armonk, NY, USA) and graphs were made using GraphPad Prism version 7 for Windows (GraphPad Software, San Diego, CA, USA). (Two-sided) P values ≤ 0.05 were considered statistically significant. Responses to PfRBC were assessed after subtraction of responses in the uRBC condition. Most baseline data was log-transformed (log_10_(value +1)) to obtain normally distributed variables, and compared with independent-samples T tests. However, Mann-Whitney U tests were used for the transcription factor and cytokine data, which remained non-normally distributed.

Linear mixed models were used to obtain fixed predicted values of follow-up data, after confirming a normal distribution of residual variables. The models were based on restricted maximum likelihood (REML) estimation and were run on log-transformed data. To compare infected and uninfected children over time as in Supplementary Figure S1, a model was used with current malaria infection status and time point as fixed effects. The time points were baseline, 9 months, and 21 months. Subject was included as a random effect with unstructured covariance type. For the analysis of time free of infection as in Fig. 1b, a model was used with time uninfected as fixed effect, and subject as a random effect with compound symmetry as covariance type. For this model, it was assumed that when a child was negative at two consecutive time points, it had been uninfected throughout this time period. For the comparison of current and previous malaria status as in Fig. 1c, a model was used with current malaria status and previous malaria status as fixed effects, and subject as a random effect with compound symmetry as covariance type.

### Data availability

The datasets analysed during the current study are available from the corresponding author on reasonable request.

## Electronic supplementary material

Supplementary Information
