# Supplementary material for: Longitudinal study of changes in γδ T cells and CD4+ T cells upon asymptomatic malaria infection in Indonesian children
Source: Sci Rep. 2017 Aug 18;7:8844. doi: 10.1038/s41598-017-09099-z (PMC5562820; doi:10.1038/s41598-017-09099-z)
Supplement: Supplementary file 1 — Supplementary Information [file 41598_2017_9099_MOESM1_ESM.pdf]

Supplementary Information

Longitudinal study of changes in γδ T cells and CD4+ T cells upon asymptomatic malaria infection in Indonesian children

Sanne E. de Jong[1], Vera E. R. Asscher[1], Linda J. Wammes[1,6], Aprilianto E. Wiria[1,2], Firdaus Hamid[1,3], Erliyani Sartono[1], Taniawati Supali[2], Hermelijn H. Smits[1], Adrian J. F. Luty[4,5], Maria Yazdanbakhsh[1]

[1]Leiden Immunoparasitology Group, Department of Parasitology, Leiden University Medical Center, Albinusdreef 2, 2333 ZA, Leiden, The Netherlands. [2]Department of Parasitology, University of Indonesia, Jl. Salemba Raya No. 6, 10430, Jakarta Pusat, Indonesia. [3]Department of Microbiology, Hasanuddin University, Jl. Perintis Kemerdekaan, Km. 10, 90245, Makassar, Indonesia. [4]Mère et Enfant Face aux Infections Tropicales, UMR 216, French National Research Institute for Sustainable Development (IRD), Paris, France. [5]Faculty of Pharmaceutical and Biological Sciences of Paris, Paris Descartes University, Paris, France. [6]Present address: Department of Medical Microbiology & Infectious Diseases, Erasmus MC, Wytemaweg 80, 3015 CN, Rotterdam, The Netherlands. Sanne E. de Jong and Vera E. R. Asscher contributed equally to this work. Correspondence and requests for materials should be addressed to M.Y. (email: myazdanbakhsh@lumc.nl).

Supplementary Figures

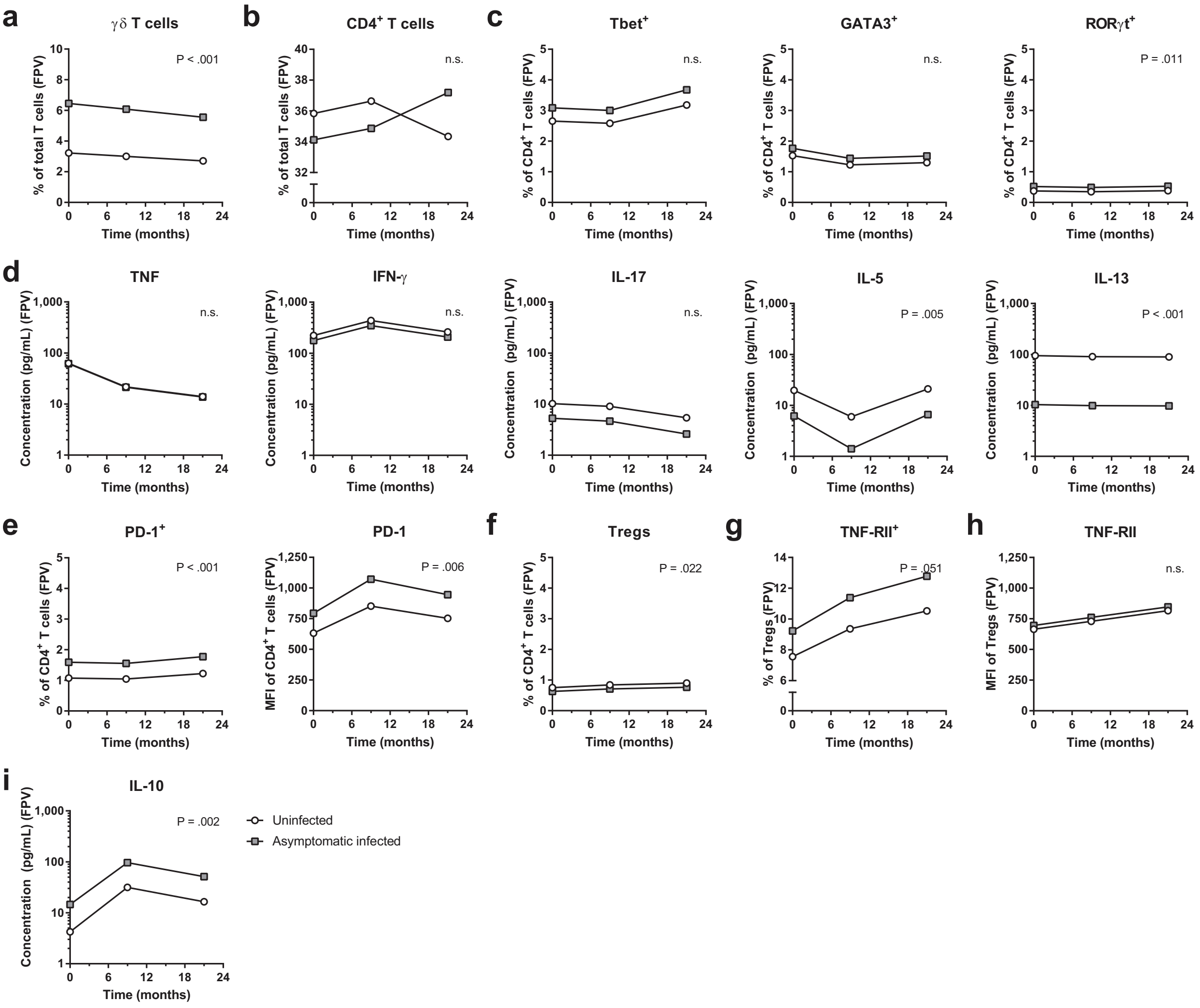

Supplementary Figure S1. Fixed predicted values (FPV) from linear mixed model analyses for cell percentages and expression levels compared between uninfected and asymptomatic infected children at baseline, 9 months, and 21 months.

The P value indicates whether the uninfected and infected children were significantly different. n.s., not significant.

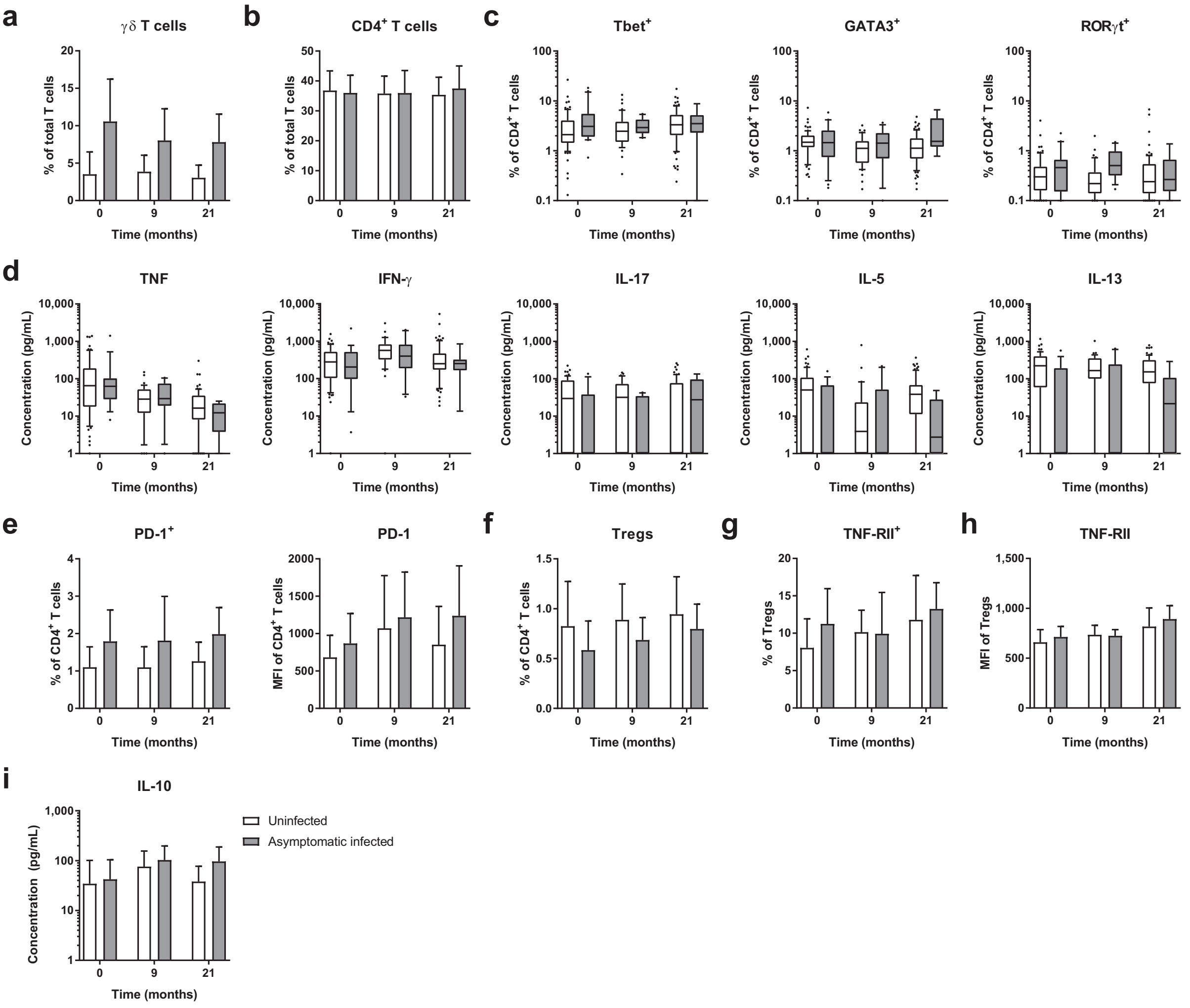

Supplementary Figure S2. Raw data of cell percentages and expression levels compared between uninfected and asymptomatic infected children at baseline, 9 months, and 21 months.

Bar graphs show mean and SEM and boxplots have 10%-90% whiskers.

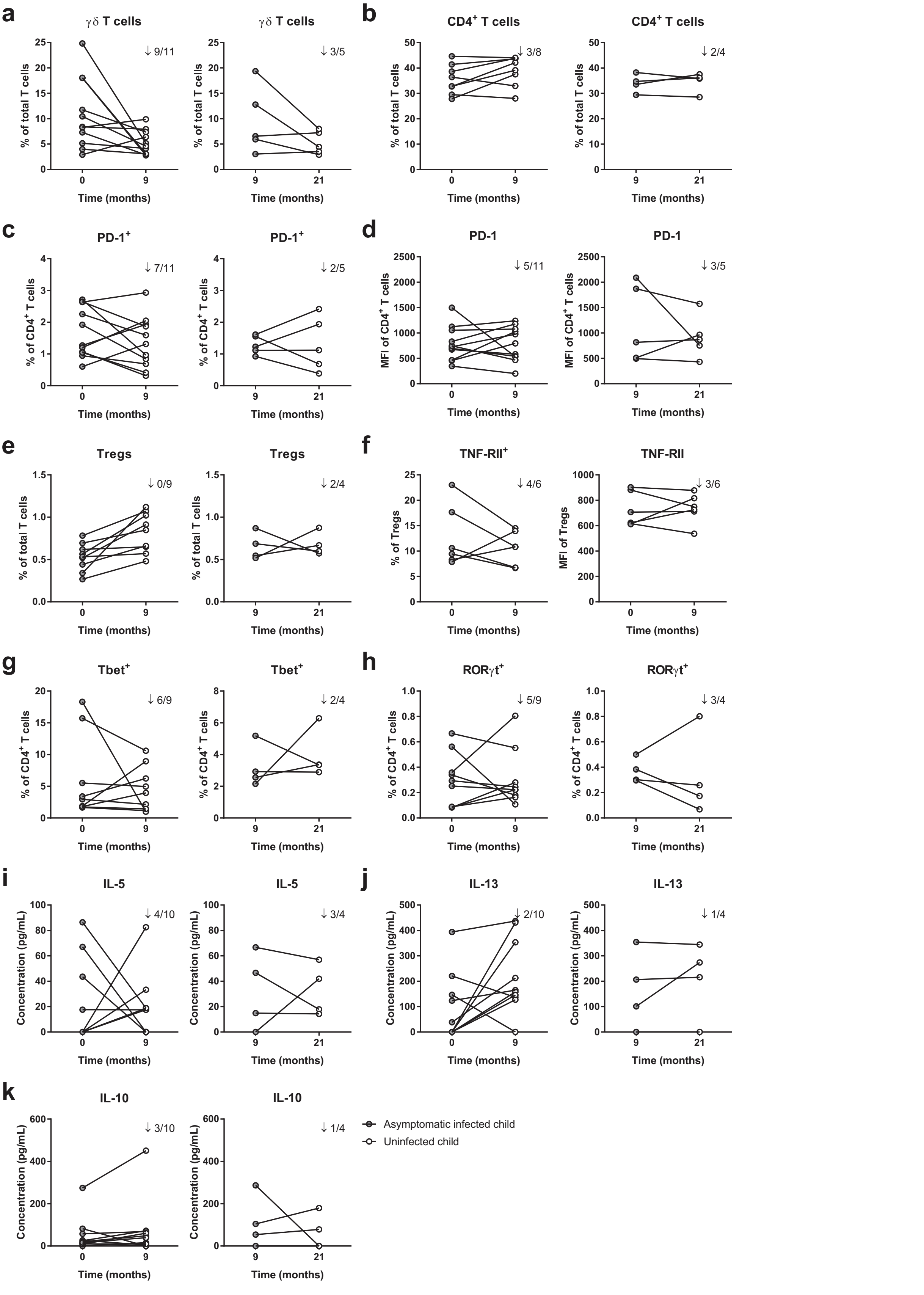

Supplementary Figure S3. Raw cell frequency, MFI, and cytokine data per child that turns from asymptomatic infected to uninfected.

The number in the top right shows how for how many children the value decreases over time.

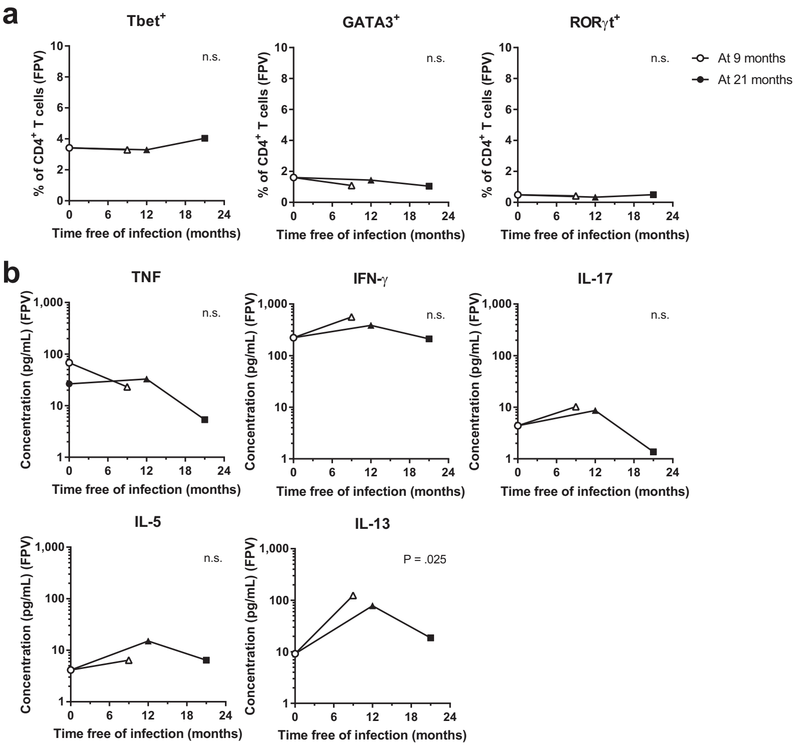

Supplementary Figure S4. Analyses of the longevity of differences between asymptomatic infected and uninfected children for T cell subsets and cytokine responses.

a) The percentage of $Tbet^+$ (Th1), $GATA3^+$ (Th2), and $ROR\gamma t^+$ (Th17) cells amongst $CD4^+$ T cells in children uninfected at either the 9 or 21 months' time point were plotted against the time period the children had been free of infection at the respective time point. Data shown are fixed predicted values (FPVs) of linear mixed modelling. b) The concentration of TNF, IFN-γ, IL-17, IL-5, and IL-13 detected in supernatant after PfRBC stimulation compared between children who had been free of infection for longer and shorter time periods.

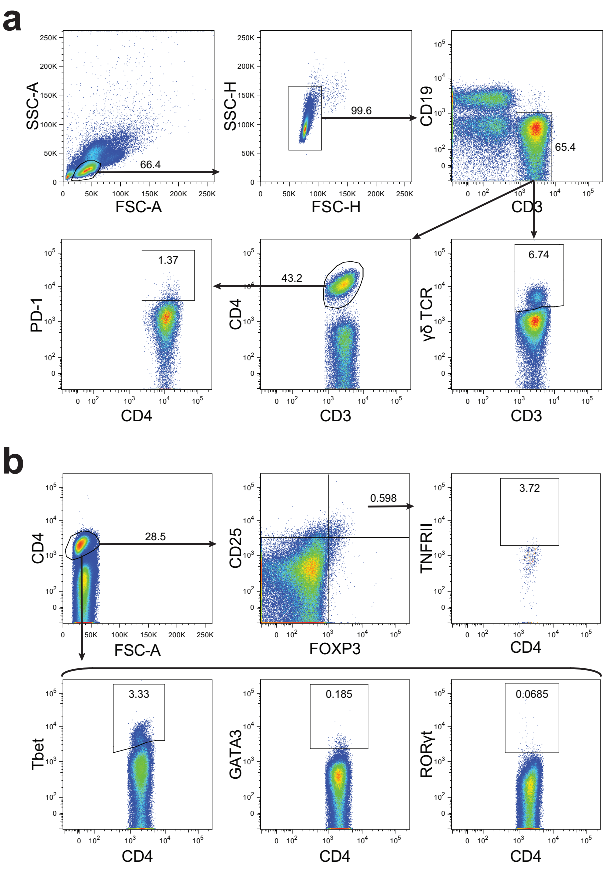

Supplementary Figure S5. FlowJo gating strategies.

a) Gating strategy as used for panel 1. γδ T cells were gated from lymphocytes, then on single cells (singlets), then on CD3$^+$ CD19$^-$ cells, and finally on γδTCR$^+$ cells. PD-1 expression was determined on CD4$^+$ T cells gated from lymphocytes, then on singlets, then on CD3$^+$ CD19$^-$ cells, and finally on CD4$^+$ cells. b) Gating strategy as used for panel 2 and 3. Tregs were gated from lymphocytes, then on singlets, then on CD4$^+$ cells, and finally on CD25^{Hi} FOXP3$^+$ cells. TNF-RII expression was gated on Tregs. Tbet, GATA3 and RORγt expression of CD4$^+$ T cells was determined on cells gated from lymphocytes, then on singlets, and finally on CD4$^+$ cells.

Supplementary Tables

Supplementary Table S1. Prevalence of *Plasmodium* infection in the study population.

Time point	*Plasmodium* species*	n
Baseline (N=123)	Any *Plasmodium*	27 (22.0%)
	Pf single infection	15 (55.5%)
	Pv single infection	8 (29.6%)
	Pm single infection	1 (3.7%)
	Pf + Pv co-infection	1 (3.7%)
	Pf + Pm co-infection	2 (7.4%)
9 months (N=77)	Any *Plasmodium*	17 (22.1%)
	Pf single infection	7 (41.2%)
	Pv single infection	3 (17.6%)
	Pm single infection	2 (11.8%)
	Pf + Pv co-infection	5 (29.4%)
21 months (N=115)	Any *Plasmodium*	10 (8.7%)
	Pf single infection	3 (30.0%)
	Pv single infection	4 (40.0%)
	Pm single infection	1 (10.0%)
	Pf + Pv co-infection	2 (20.0%)

*Pf, *Plasmodium falciparum*. Pv, *Plasmodium vivax*. Pm, *Plasmodium malariae*.

Supplementary Table S2. Helminth prevalence in the study population.

Time point	Helminth infection (%)	All children	*Plasmodium* uninfected children	*Plasmodium* infected children	P-value*
Baseline	Any helminth	93.3	94.3	89.5	.604
	Trichuris (microscopy)	49.0	49.4	47.8	1.000
	Any helminth (PCR)	84.6	83.3	89.5	.726
	A. lumbricoides (PCR)	51.6	50.0	57.9	.611
	S. stercoralis (PCR)	0.0	0.0	0.0	1.000
	Any hookworm (PCR)	79.1	76.4	89.5	.342
	A. duodenale (PCR)	11.0	12.5	5.3	.459
	N. americanus (PCR)	78.0	75.0	89.5	.225
9 months	Any helminth	69.6	70.4	62.5	.760
	Trichuris (microscopy)	41.4	41.8	47.1	.783
	Any helminth (PCR)	59.3	58.2	62.5	.783
	A. lumbricoides (PCR)	31.0	41.8	43.8	1.000
	S. stercoralis (PCR)	3.5	0.0	12.5	.048
	Any hookworm (PCR)	45.1	38.2	37.5	1.000
	A. duodenale (PCR)	5.3	1.8	6.3	.402
	N. americanus (PCR)	45.1	38.2	37.5	1.000
21 months	Any helminth	74.3	75.6	55.6	.237
	Trichuris (microscopy)	40.6	42.2	33.3	.732
	Any helminth (PCR)	55.4	55.6	44.4	.728
	A. lumbricoides (PCR)	26.7	27.8	22.2	1.000
	S. stercoralis (PCR)	1.0	0.0	11.1	.091
	Any hookworm (PCR)	41.6	41.1	33.3	.736
	A. duodenale (PCR)	2.0	1.1	0.0	1.000
	N. americanus (PCR)	41.6	41.1	33.3	.736

* Comparing *Plasmodium*-uninfected children with asymptomatically infected children by Mann-Witney test.

Supplementary Table S3. Antibody panels used for flow cytometry.

Panel	Channel	Fluorochrome	Specificity	Clone	Vendor	Cat. no.	Dilution
1	FL3	PerCP-eFluor710	PD-1 (CD279)	J105	eBioscience	46-2799	400x
	FL4	PE-Cy7	CD4	SK3	BD Biosciences	557852	100x
	FL6	APC-eFluor780	CD3	UCHT1	eBioscience	47-0038	800x
	FL7	Pacific Blue	CD19	HIB19	BioLegend	302224	200x
	FL8	Biotin	[illegible]TCR	B1.1	eBioscience	13-9959	80x
		Qdot525	Streptavidin	-	Invitrogen	Q10141MP	150x
2	FL2	PE	ROR[illegible]t	AFKJS-9	eBioscience	12-6988	150x
	FL3	PerCP-Cy5.5	T-bet	4B10	eBioscience	45-5825	320x
	FL4	PE-Cy7	CD25	2A3	BD Biosciences	335824	160x
	FL5	eFluor660	GATA3	TWAJ	eBioscience	50-9966	80x
	FL6	APC-Cy7	CD4	RPA-T4	eBioscience	47-0049	400x
	FL7	eFluor450	FOXP3	PCH101	eBioscience	48-4776	100x
3	FL4	PECy7	CD25	2A3	BD Biosciences	335824	160x
	FL5	APC	FOXP3	PCH101	eBioscience	17-4776	100x
	FL6	APC-Cy7	CD4	RPA-T4	eBioscience	47-0049	400x
	FL8	Biotin	TNF-RII (CD120b)	MR2-1	Hycult Biotech	HM2008	50x
		Qdot525	Streptavidin	-	Invitrogen	Q10141MP	150x

Supplementary Table S4. BD FACSCanto II flow cytometer specifications.

Laser	Detector	Mirror	Filter	Channel
Violet (405 nm)	A	502 LP	510/50 BP	FL8
	B		450/50 BP	FL7
Blue (488 nm)	A	735 LP	780/60 BP	FL4
	B	655 LP	670 LP	FL3
	C	610 LP		-
	D	556 LP	585/42 BP	FL2
	E	502 LP	530/30 BP	FL1
	F		488/10 BP	SSC
Red (633 nm)	A	735 LP	780/60 BP	FL6
	B	685 LP		-
	C		660/20 BP	FL5
